# Risk Factors for Falls in Adults With Rheumatoid Arthritis: A Prospective Study

**DOI:** 10.1002/acr.21987

**Published:** 2013-07-26

**Authors:** Emma K Stanmore, Jackie Oldham, Dawn A Skelton, Terence O'Neill, Mark Pilling, A John Campbell, Chris Todd

**Affiliations:** 1 University of Manchester and Manchester Academic Health Science CentreManchester, UK; 2 Arthritis Research UK and University of ManchesterManchester, UK; 3 Glasgow Caledonian UniversityGlasgow, UK; 4 Dunedin School of MedicineDunedin, New Zealand

## Abstract

***Objective:*** To investigate the association between potential risk factors and falls in community-dwelling adults with rheumatoid arthritis (RA).

***Methods:*** We followed patients for 1 year of followup in a prospective cohort study with monthly falls calendars and telephone calls. Lower extremity muscle strength, postural stability, number of swollen and tender joints, functional status, history of falling, fear of falling, pain, fatigue, medication, and use of steroids were assessed as risk factors for falls.

***Results:*** A total of 386 women and 173 men with RA (n = 559) ages 18–88 years completed baseline assessments and 535 participants (96%) completed 1-year followup. Bivariate logistic regression showed that falls risk was not associated with age or sex. Multivariate logistic regression revealed that a history of multiple falls in the previous 12 months was the most significant predictive risk factor (odds ratio [OR] 5.3, 95% confidence interval [95% CI] 2.3–12.3). The most significant modifiable risk factors were swollen and tender lower extremity joints (OR 1.7, 95% CI 1.1–2.7), psychotropic medication (OR 1.8, 95% CI 1.1–3.1), and fatigue (OR 1.13, 95% CI 1.02–1.2).

***Conclusion:*** Adults with RA are at high risk of falls. In clinical practice, high-risk fall patients with RA can be identified by asking whether patients have fallen in the past year. Important risk factors highlighted in this study include swollen and tender lower extremity joints, fatigue, and use of psychotropic medications.

## INTRODUCTION

Adults with rheumatoid arthritis (RA) have an increased risk of falls (1,2). Suggested reasons for this include impaired muscle strength, postural instability, fatigue, joint pain, and reduced functioning (2–5). The falls also lead to an increased risk of hip fractures due to disease-related reduced bone mass (5–6). Other fall consequences include serious injuries, hospital admission or admission to care homes, fear of falling, and reduced quality of life.

Estimates of the proportion of people with RA who fall annually range from 10–54% (1–12), and this high variability may be due to the sample selection (women only, small samples, or frail older patients), inconsistent definitions or no definitions of falls, and use of different assessment measures. The risk factors for falls in patients with RA that have been drawn from previous studies include tender joint count (11–12), swollen joint count (2–4), pain in lower extremities (9), pain intensity (4,7), Health Assessment Questionnaire (HAQ) disability score (4–12), low levels of physical activity (4), impaired general health (4–12), use of antidepressants (3), impaired vision (4), impairment in both walking and rising (3), walk time (8), impaired balance (2), number of medications (3), number of comorbidities (8), and 1-year history of falls (7). Some of these risk factors are common to older people (e.g., impaired vision, previous history of a fall, and number and types of medications) (13), but others appear to be disease specific (e.g., swollen or tender joints, pain, and increased HAQ disability scores).

To date, there has not been a sufficiently large prospective study of adults of all ages with RA to provide a comprehensive investigation of the fall risk factors associated with RA. The identification of predictive and potentially modifiable risk factors is essential for the development of effective falls prevention strategies. The aim of this study was to identify fall risk factors in adults with RA.

**Significance & Innovations**

- Lower extremity muscle strength, postural stability, number of swollen and tender joints, functional status, history of falling, fear of falling, pain, fatigue, medication, and use of steroids were assessed as risk factors for falls in adults with rheumatoid arthritis (RA).
- Important risk factors found in this study include swollen and tender lower extremity joints, fatigue, and use of psychotropic medications.
- Adults with RA, regardless of age, are at high risk of falls.

## SUBJECTS AND METHODS

A consecutive sample of eligible patients was recruited from 4 rheumatology clinics in the Northwest of England. A variety of different clinics were accessed to ensure people with different levels of disease severity were invited to participate. All participants were ages >18 years with a diagnosis of RA based on the 2010 American College of Rheumatology/European League Against Rheumatism classification criteria for RA (14). Participants were excluded from the study if they were age <18 years or if they did not have the mental or physical capacity to give informed consent (assessed by a research nurse). This study was conducted with the approval of the National Research Ethics Committee (reference 08/H1009/41).

Measurements were taken at baseline between the months of August 2008 and March 2009, and participants were followed up for falls and injuries for 1 year using preaddressed, prepaid daily falls calendars (posted monthly) and monthly followup telephone calls.

### Data collection

Variables considered important in causing or predicting falls were assessed at baseline by trained research nurses experienced in undertaking joint counts.

RA status was assessed by the number of swollen/tender joints (shoulders, elbows, wrists, metacarpophalangeal joints, proximal interphalangeal joints, and knees), the Disease Activity Score in 28 joints (DAS28), and the Stanford Arthritis Centre HAQ. The DAS28 has been extensively validated for use in clinical trials and practice (15). It provides scores for the number of swollen and tender joints, erythrocyte sedimentation rate, and a visual analog scale (VAS) global disease scale. The total DAS28 ranges from 0–10 and indicates the current activity of RA. Accepted cutoffs are 5.1 for high disease activity and 3.2 for low disease activity. The HAQ is a self-administered arthritis-specific instrument that measures patients' perceptions of difficulties in performing activities in daily living and the need for equipment and physical assistance to perform tasks, and has been extensively tested for validity and reliability (16).

Fear of falling was recorded using the Short Falls Efficacy Scale-International (Short FES-I). The Short FES-I is a validated and reliable 7-item tool that measures fear of falling related to a range of activities (17).

Falls risk was measured by the validated Falls Risk Assessment Tool, which includes questions on the history of any fall in the previous year, taking ≥4 prescribed medications, and diagnosis of stroke or Parkinson's disease (18). Vision was assessed using a self-reported question (which gives a score of 0–4) (19). Patients were also asked questions about levels of pain and fatigue using VAS (20–21) and about any comorbidities (number and type) and previous fractures, surgery, or joint replacement(s) (4), and verified using medical records. Medical records were also used to check history and previous medication use, including steroid use (11).

Lower extremity muscle strength and balance were assessed using the Chair Stand Test (22) and the Four-Test Balance Scale (23). For the Chair Stand Test, participants were instructed to stand up and down from a chair as quickly as possible 5 times with their arms folded. The time taken to complete was recorded. The Four-Test Balance Scale comprised 4 timed static balance tasks of increasing difficulty using different positioning of the participants' feet. Participants were scored 0 for unsuccessful, 1 if they could only stand with their feet together, 2 if they could only complete a semitandem stand, 3 if they could complete a tandem stand, and 4 for participants who could complete a one-leg stand. The participant must hold each position for 10 seconds before progressing to the next more challenging task.

The Prevention of Falls Network Europe (ProFaNE) definition of “an unexpected event in which participants come to rest on the ground, floor, or other lower level” was used to identify falls, rather than trips or stumbles (24). Participants who reported a fall, failed to return a falls calendar, or filled in the calendar incorrectly were contacted by telephone each month. The methods of Campbell et al were used to collect information about the fall event during the followup telephone calls (25), as recommended by Schwenk and colleagues (26). Data included the date of the fall, a self-reported description of how the fall occurred, consequences and injuries, and health care utilization (e.g., hospital admission, medical assistance, physiotherapy).

### Statistical analysis

The ProFaNE consensus group–recommended guidance on fall data analysis was utilized for analysis of the data (24). Analysis of variance (ANOVA) was used to test for differences between the groups of nonfallers, single fallers, and multiple fallers. Levene's test for homogeneity of variance was initially applied (27). When homogeneity of variances was met (*P* greater than 0.05), ANOVA was undertaken and Tukey's post hoc tests were used to compare the differences between pairs of groups. In cases when Levene's test was not met (*P* less than or equal to 0.05), Welch's test (27) was used to determine overall significance between the groups, and Dunnett's T3 post hoc tests (27) were used to compare between pairs of groups.

Chi-square tests of trend were used as appropriate for categorical data to examine differences in groups of nonfallers, single fallers, and multiple fallers. Binary logistic regression was used to calculate odds ratios (ORs) and 95% confidence intervals (95% CIs) for age, sex, and all fall risk–associated variables, with occurrence of falls during the study as the outcome. Variables were initially examined using bivariate analyses to estimate associations for each risk factor with fall outcomes. To avoid an underpowered logistic regression analysis, the 3 groups (nonfallers, single fallers, and multiple fallers) were combined into 2 groups (nonfallers and all fallers) and a limited number of explanatory variables were selected based on statistical significance (*P* less than 0.05). These variables were selected using the Hosmer-Lemeshow approach (28). The selected variables were entered into 2 multivariate analyses, using binary multivariate logistic regression to build predictive and explanatory risk models. In addition to understanding the best predictive risk factors, it is clinically important to understand the risk factors that can potentially be modified to enable an effective falls prevention strategy to be implemented. Therefore, an explanatory risk factor model excluding a 12-month history of falls as well as a predictive risk factor model were added to the analysis to explore the potentially modifiable reasons for falls. Multicollinearity was assessed using a variance inflation factor (VIF) >10. Statistical analysis was performed using SPSS, version 16.0 (29).

## RESULTS

### Subject characteristics

The baseline characteristics of the sample are provided in Table1. The mean ± SD age of the male participants was 62 ± 11.0 years and the mean ± SD age of the female participants was 61.9 ± 13.6 years. There were more than twice as many women (n = 386 [69%]) as men recruited to the study and 68% of the participants were married (n = 378). The majority of the participants described themselves as white and British (n = 544 [97%]). More than half were retired from employment (n = 327 [59%]), with only one-quarter in employment (n = 134 [24%]). The mean DAS28 score of the participants (4.1, mode 3.9) fell within the moderate disease activity range (3.3–5.1). The majority of the participants had comorbidities, with hypertension (n = 149 [27%]), respiratory disease (n = 92 [16%]), cardiovascular disease (n = 82 [15%]), and osteoarthritis (n = 78 [14%]) being the most common. There were 19 variables used in the analysis, of which there were a total of 16 missing values among the 535 participants, leading to a missing data rate of 0.18%.

**Table 1:** Baseline demographics and 1-year prestudy characteristics*

	Total (n = 535)	Nonfaller group (n = 340)	Single faller group (n = 94)	Multiple faller group (n = 101)	Overall *P*
Age, mean ± SD years	62 ± 13.6	62 ± 12.7	66 ± 11.7	61 ± 12.7	0.12†
Women, no. (%)	386 (69.1)	235 (69.1)	68 (72.3)	70 (69.3)	0.83‡
No. of swollen joints (range 0–28), mean ± SD	4.7 ± 6.3	4.5 ± 6.1	3.8 ± 5.0	5.8 ± 7.0	0.09†
No. of tender joints (range 0–28), mean ± SD	5.3 ± 6.9	5.0 ± 6.6	4.2 ± 5.5	7.0 ± 8.0	0.02†
DAS28 score (range 0–10), mean ± SD	4.1 ± 1.6	3.9 ± 1.6	4.1 ± 1.3	4.5 ± 1.5	0.002†
Use of psychotropic medication, no. (%)	105 (18.8)	47 (13.8)	20 (21.3)	34 (33.7)	< 0.001‡
Taking ≥4 types of medications each day, no. (%)	431 (77.1)	247 (72.4)	83 (88.3)	82 (81.2)	0.003‡
Taking steroids at baseline, no. (%)	117 (20.9)	61 (17.9)	21 (22.3)	28 (27.7)	0.03‡
History of stroke or Parkinson's disease, no. (%)	38 (6.8)	18 (5.3)	5 (5.3)	13 (12.9)	0.02‡
VAS pain score (range 0–10), mean ± SD	3.85 ± 2.7	3.5 ± 2.6	3.8 ± 2.6	5.0 ± 2.4	< 0.001†
VAS fatigue score (range 0–10), mean ± SD	4.7 ± 2.8	4.2 ± 2.7	5.2 ± 2.9	5.8 ± 2.2	< 0.001†
History of fall in previous 12 months, no. (%)	232 (43.4)	108 (31.8)	53 (56.4)	71 (70.3)	< 0.001‡
History of no falls in previous 12 months, no. (%)	303 (56.6)	232 (68.2)	41 (43.6)	30 (29.7)	–
History of single fall in previous 12 months	116 (21.7)	58 (17.1)	36 (38.3)	22 (21.8)	–
History of multiple falls in previous 12 months	116 (21.7)	50 (14.7)	17 (18.1)	49 (48.5)	–
History of fractures, no. (%)	228 (40.8)	127 (37.4)	39 (41.5)	53 (52.5)	0.008‡
History of injuries from previous falls (range 0–6), mean ± SD	1.6 ± 1.5	–	1.8 ± 1.1	2.5 ± 1.4	< 0.001†
Poor vision (registered blind, very poor, or poor), no. (%)	46 (8.6)	26 (7.6)	8 (8.5)	12 (11.9)	0.87‡
No. of comorbidities (range 0–10), mean ± SD	2.0 ± 1.9	1.9 ± 1.9	2.1 ± 1.9	2.2 ± 2.3	0.36†
Previous surgery, no. (%)	408 (73.1)	246 (72.4)	66 (70.2)	79 (78.2)	0.63‡
Painful feet, no. (%)	432 (77.3)	260 (76.5)	72 (76.6)	84 (83.2)	0.17‡
No. of joint replacements (range 0–4), no. (%)	125 (22.5)	76 (22.5)	22 (23.4)	24 (23.8)	0.74‡
Symptoms of feeling dizzy or unsteady, no. (%)	370 (66.2)	209 (61.5)	64 (68.1)	80 (79.2)	0.01‡
Fear of falling: Short FES-I score (range 7–28), mean ± SD	15.3 ± 6.5	14.4 ± 6.4	15.6 ± 17.8	17.8 ± 5.6	> 0.01†
HAQ score (range 1–4), mean ± SD	2.4 ± 0.9	2.3 ± 0.8	2.5 ± 0.8	2.8 ± 0.8	< 0.001†
Fail at each level of the Four-Test Balance Scale, no. (%)					
Unsuccessful (0)	39 (7.0)	19 (5.6)	6 (6.4)	11 (10.9)	–
Feet together stand (1)	13 (2.2)	5 (1.5)	2 (2.1)	3 (3.0)	–
Semitandem stand (2)	216 (38.6)	127 (37.4)	42 (44.7)	38 (37.6)	–
Tandem stand (3)	116 (20.8)	67 (19.7)	20 (21.3)	25 (24.8)	–
One-leg stand (4)	175 (31.3)	122 (35.9)	24 (25.5)	24 (23.8)	0.008‡
Ability to complete 5 chair stands, no. (%)	484 (86.6)	307 (90.3)	83 (88.3)	76 (75.2)	< 0.001‡
Time taken to perform 5 chair stands, mean ± SD seconds	20.9 ± 12.2	19.8 ± 11.2	22.8 ± 14.1	24.2 ± 13.7	0.02†

* DAS28 = Disease Activity Score in 28 joints; VAS = visual analog scale; Short FES-I = Short Falls Efficacy Scale-International; HAQ = Health Assessment Questionnaire.

† Significant differences were evaluated by one-way analysis of variance.

‡ Significant differences were evaluated by the chi-square test.

Among the 535 participants with RA, 195 (36%; 95% CI 32–41%) reported a fall during the 1-year followup. In the 1-year followup, there were 340 nonfallers (64%), 94 single fallers (those who fell once, 18%), and 101 multiple fallers (those who fell more than once, 19%). In the year preceding entry to the study, there were 317 nonfallers (57%), 120 single fallers (21%), and 122 multiple fallers (22%). The probability of a 1-year followup fall significantly increased (*P* < 0.001) if the participant fell during the previous 12 months. Of those with a 1-year history of falls, 124 (53.4%) reported a 1-year followup fall, whereas of those with no 1-year history of falls, only 71 (23.4%) reported study falls.

Multiple fallers had a significantly higher mean Short FES-I score than single fallers (mean difference 2.2; *P* = 0.03) and nonfallers (mean difference 3.5; *P* < 0.001). There were no significant differences between the mean Short FES-I score in the groups of single fallers and nonfallers (mean difference 1.3; *P* = 0.19). These results suggest that multiple fallers have significantly higher levels of fear of falling compared to single fallers or nonfallers; however, in clinical terms, these differences are small.

VAS pain scores were significantly higher in multiple fallers (mean difference 1.5; 95% CI 0.83–2.20, *P* < 0.001) than in nonfallers and single fallers (mean difference 1.1; 95% CI 0.28–2.01, *P* = 0.006). However, there were no significant differences between single fallers and nonfallers in baseline VAS pain scores (mean difference 0.4; *P* = 0.44).

VAS fatigue scores were significantly higher in single fallers (mean difference 1.1; *P* = 0.005) and multiple fallers (mean difference 1.6; *P* < 0.001) than in nonfallers. However, there were no significant differences between single fallers and multiple fallers in VAS fatigue scores (mean difference 0.6; *P* = 0.34).

Mean DAS28 scores were significantly higher in multiple fallers (mean difference 0.6; *P* = 0.001) than in nonfallers. However, there were no significant differences between single fallers and multiple fallers (mean difference 0.5; *P* = 0.07) and between single fallers and nonfallers in DAS28 scores (mean difference 0.2; *P* = 0.61).

Single fallers had a higher mean HAQ score than nonfallers (mean difference 0.2; *P* = 0.06), and this was borderline significant. Multiple fallers had a significantly higher mean HAQ score than single fallers (mean difference 0.3) and nonfallers (mean difference 0.5; *P* = 0.04).

Compared to nonfallers and single fallers at followup, at baseline, those experiencing multiple falls were more likely to take ≥4 types of medications (*P* = 0.013), receive psychotropic medications (*P* < 0.001), report feeling dizzy or unsteady (*P* = 0.01), and have a history of stroke or Parkinson's disease (*P* = 0.02); were less likely to be able to complete the Chair Stand Test than nonfallers (*P* < 0.001); and were less likely to be able to complete the semitandem stand, the tandem stand, or the one-leg stand (*P* = 0.008), take steroid medication (*P* = 0.03), or have a history of fracture (*P* = 0.008) (Table1).

### Risk factors

The results comparing all fallers with nonfallers using logistic regression analyses are shown in Table2. The variables were classified into groups of demographic, medical, self-report/functional ability, and postural risk factors.

**Table 2:** Associations between fall risk factors and fallers using bivariate binary logistic regression (all fallers n = 195/nonfallers n = 340)*

	OR (95% CI)
Demographic risk factors	
Sex	
Male	Referent
Female	1.1 (0.7–1.6)
Age (range 18–88 years)	1.0 (0.99–1.02)
Medical risk factors	
No. of tender joints (range 0–28)	1.0 (0.98–1.04)
Swollen or tender lower extremity joints	
No	Referent
Yes	2.0 (1.3–2.8)
DAS28 score (range 0.1–8)	1.2 (1.1–1.3)
Use of psychotropic medications	
No	Referent
Yes	2.4 (1.5–3.7)
Taking ≥4 types of medications	
No	Referent
Yes	2.1 (1.3–3.3)
Taking steroids at baseline	
No	Referent
Yes	1.5 (1.0–2.4)
History of stroke or Parkinson's disease	
No	Referent
Yes	1.8 (0.9–3.6)
No. of comorbidities (range 0–10)	1.0 (0.97–1.2)
VAS pain score (range 0–10)	1.2 (1.1–1.2)
VAS fatigue score (range 0–10)	1.2 (1.1–1.3)
History of falls in previous 12 months	
0 falls	Referent
1 fall	3.3 (2.1–5.1)
≥2 falls	4.3 (2.7–6.8)
History of fracture	
No	Referent
Yes	1.5 (1.04–2.1)
History of injuries from previous falls (range 0–6)	1.3 (1.1–1.6)
Self-report/functional ability risk factors	
Short FES-I score (range 7–28)	1.1 (1.03–1.1)
HAQ score (range 1.00–4.00)	1.7 (1.4–2.1)
Postural risk factors	
Four-Test Balance Scale	
Unsuccessful (0)	2.3 (1.1–4.7)
Feet together stand (1)	2.5 (0.7–9.1)
Semitandem stand (2)	1.6 (1.0–2.5)
Tandem stand (3)	1.7 (1.0–2.8)
One-leg stand (4)	Referent
Symptoms of feeling dizzy or unsteady	
No	Referent
Yes	1.8 (1.2–2.6)
Ability to complete the Chair Stand Test	
No	Referent
Yes	0.48 (0.29–0.8)
Time taken for the Chair Stand Test (n = 484, range 4–104 seconds)	1.02 (1.01–1.04)

* OR = odds ratio; 95% CI = 95% confidence interval; DAS28 = Disease Activity Score in 28 joints; VAS = visual analog scale; Short FES-I = Short Falls Efficacy Scale-International; HAQ = Health Assessment Questionnaire.

#### Medical risk factors

There were no associations found between the number of tender joints and falls. Reporting any swollen or tender lower extremity joints (hip, knee, or ankle; feet not included) doubled the risk of falling during the followup period. The DAS28 score was another predictor of falls that could be useful in clinical practice (OR 1.2). Taking psychotropic medications more than doubled the odds of falling. Polypharmacy was a significant predictor of falls; taking ≥4 medications more than doubled the risk of falling. Taking steroids at baseline increased the risk of falling by half, as did a history of previous fracture(s). There were no associations found between a history of stroke or Parkinson's disease and falls. Both the VAS pain and VAS fatigue scores showed similar positive predictive values for falling, with the risk raised by 20% for every 1-point increase in the score. A positive self-reported history of falls in the previous 12 months at baseline was a strong predictor of falls. Reporting a single fall in the previous 12 months at baseline more than tripled the risk of falling during the reporting period of our prospective study, and reporting multiple falls more than quadrupled the risk. Reporting 12-month previous injurious falls at baseline (OR 1.3) and a history of fracture(s) (OR 1.5) were also strong predictors of falls.

#### Self-report/functional ability risk factors

The Short FES-I score values range from 7 (no fear of falling) to 28 (very fearful of falling), and for each 1-unit increase there was a 10% increase in odds of falls. The bivariate analyses demonstrated that for each additional point attained in the final HAQ score (range 1.00–4.00), the risk of falling increased by 70%.

#### Postural risk factors

The ORs between the groups in the Four-Test Balance Scale increased as the groups became more impaired. The odds of falling was 2.3 times higher for participants who could not complete the Four-Test Balance Scale at all and 2.5 times higher for those who could only complete the feet together stand; however, the 95% CIs spanned agreement in this scale, possibly due to smaller numbers of participants. A symptom of feeling dizzy or unsteady was also a strong predictor of falls with participants, with positive reports having an 80% greater risk of falling than those without.

There was an association found with those who were able to complete the Chair Stand Test with an OR of 0.48, which means that there was a protective association for falls over the 1-year followup for those who were able to complete the test. Therefore, those able to complete the Chair Stand Test were half as likely to fall as those unable to complete the test. The time taken to complete the Chair Stand Test varied from 4–104 seconds. For every additional second taken to complete the test, there was an increased risk of falling of 2%.

### Multivariate analysis of predictive risk factors

Multivariate logistic regression was used to build a predictive model that could be useful to gauge fall risk. Due to the limited number of participants who fell (n = 195), only a selected number of variables could be included in a multivariate regression in order to avoid model overspecification. The following variables were included in this multivariate analysis based on their statistical significance in the bivariate analysis: swollen or tender lower extremity joints, taking psychotropic medications, taking ≥4 medications, a history of fracture or injuries, the ability and time taken to complete the Chair Stand Test, the ability to complete the Four-Test Balance Scale, feeling dizzy or unsteady, fear of falling (Short FES-I score), history of a single fall, history of multiple falls, DAS28 score, taking steroids, pain, fatigue, and HAQ score. Multivariate logistic regression analysis initially showed that the results from the ability to complete the Chair Stand Test variable were highly correlated with the other variables, causing spurious model estimates (VIF >10) (22). Therefore, the ability to complete the Chair Stand Test variable was excluded from the analysis. The same variables (excluding the ability to complete the Chair Stand Test but not the time to complete the Chair Stand Test) were entered into multivariate logistic regression analyses to build the best predictive model of falls. The results from the multivariate analysis for predictive risk factors are shown in Table3.

**Table 3:** Results from a multivariate analysis based on 16 predictive risk factors of all fallers (n = 195) versus nonfallers (n = 340)*

	OR (95% CI)
Swollen or tender lower extremity joints	1.7 (1.1–2.8)†
DAS28 score (range 0.1–8)	0.9 (0.8–1.1)
Use of psychotropic medications (yes/no)	1.6 (0.9–2.9)
Taking ≥4 types of medications (yes/no)	1.8 (1.5–3.1)†
Taking steroids at baseline (yes/no)	1.3 (0.8–2.3)
VAS pain score (range 0–10)	1.02 (0.9–1.1)
VAS fatigue score (range 0–10)	1.11 (1.0–1.3)†
12-month history of a single fall (yes/no)	3.6 (1.8–7.3)†
12-month history of multiple falls (yes/no)	5.3 (2.3–12.3)†
A history of fracture (yes/no)	1.3 (0.8–1.9)
A history of injuries from previous falls (yes/no)	0.8 (0.6–1.1)
Short FES-I score (range 7–28)	1.0 (0.9–1.0)
HAQ score (range 1.00–4.00)	1.2 (0.7–2.0)
Four-Test Balance Scale (range 0–4)	1.0 (0.8–1.3)
Symptoms of feeling dizzy or unsteady (yes/no)	0.9 (0.5–1.5)
Time taken to complete the Chair Stand Test, seconds	0.99 (0.98–1.02)

* OR = odds ratio; 95% CI = 95% confidence interval; DAS28 = Disease Activity Score in 28 joints; VAS = visual analog scale; Short FES-I = Short Falls Efficacy Scale-International; HAQ = Health Assessment Questionnaire.

† Significant.

### Predictive risk model

The final model included all 16 selected risk factor variables in predicting the occurrence of falls during the study, and accurately explained 71% of the variation in the data. Statistically significant variables were the 12-month history of a single fall (OR 3.6, 95% CI 1.8–7.3; *P* < 0.001) or multiple falls (OR 5.3, 95% CI 2.3–12.3; *P* < 0.001), swollen or tender lower extremity joints (OR 1.7, 95% CI 1.1–2.8; *P* = 0.02), increasing VAS fatigue (OR 1.11, 95% CI 1.0–1.3; *P* = 0.03), and taking ≥4 types of medications (OR 1.8, 95% CI 1.5–3.1).

### Explanatory risk factor model

A multivariate logistic regression analysis of the risk factors excluding a 12-month history of fall(s) was conducted to explore the potential reasons for falls. In addition to understanding the best predictive risk factors, it is clinically important to understand the risk factors that can potentially be modified to enable an effective falls prevention strategy to be implemented. Although a history of a single fall or multiple falls was found to be the best independent predictor of falls, this model does not help clinicians to prevent the initial fall, and a history of a single fall or multiple falls can be considered a marker of poor mobility or frailty (30). Therefore, a history of a single fall or multiple falls was excluded from the analysis due to its lack of utility in designing an intervention. The 12 variables included in the multivariate logistic regression were chosen in advance from the 18 significant variables examined in the bivariate analysis. DAS28 and VAS pain scores were included as covariates and swollen or tender lower extremity joints, taking ≥4 medications, HAQ score, Short FES-I score, use of psychotropic medications, taking steroids at baseline, time taken to complete the Chair Stand Test, the Four-Test Balance Scale, symptoms of feeling dizzy or unsteady, and the VAS fatigue score were also included as the most clinically relevant for purposes of intervention. The multivariate analysis for explanatory risk factors is shown in Table4.

**Table 4:** Results from a multivariate analysis based on 12 explanatory risk factors of all fallers (n = 195) versus nonfallers (n = 340) excluding history of falls*

	OR (95% CI)
Swollen or tender lower extremity joints	1.7 (1.1–2.7)†
DAS28 score	0.9 (0.8–1.1)
Use of psychotropic medications	1.8 (1.1–3.1)†
Taking ≥4 types of medications	1.6 (0.96–2.8)
Taking steroids at baseline	1.2 (0.7–2.1)
VAS pain score	1.02 (0.92–1.1)
VAS fatigue score	1.13 (1.02–1.2)†
Fear of falling: Short FES-I score	1.004 (0.95–1.06)
HAQ score	1.11 (0.7–1.8)
Four-Test Balance Scale	1.0 (0.8–1.3)
Symptoms of feeling dizzy or unsteady	1.1 (0.7–1.7)
Time taken for the Chair Stand Test	1.002 (0.08–1.02)

* OR = odds ratio; 95% CI = 95% confidence interval; DAS28 = Disease Activity Score in 28 joints; VAS = visual analog scale; Short FES-I = Short Falls Efficacy Scale-International; HAQ = Health Assessment Questionnaire.

* Significant.

### Explanatory risk model

The multivariate logistic regression analysis for the explanatory fall risk factors showed that having any swollen or tender lower extremity joints (hip, knee, and ankle), taking psychotropic medications, and increasing VAS fatigue produced the best fitting risk factor model. The amount of variation explained by the explanatory risk factor model due to 12 variables was 68%.

## DISCUSSION

In this study, 36% of participants ages ≥18 years reported falling at least once in the 1-year followup period. This is slightly higher than the 30% reported by older people ages ≥65 years living in the community (13–31). Due to the high risk of falls and the associated increased risk of fractures, it is important to highlight factors that may be modified to prevent falls in this group.

Falls in adults with RA are not just random events, but may be predicted and possibly prevented by assessing and treating a number of independent risk factors. Asking for a history of falls will highlight those at high risk of further falls, followed by the assessment of swollen and tender lower extremity joints (hip, knee, or ankle), taking psychotropic medications, and VAS fatigue levels. We suggest that targeting interventions toward these risk factors could reduce the burden of falls in patients with RA; however, further studies are required to confirm this. Patients should be prescribed psychotropic medications with caution, with regular reviews, and should take them no longer than necessary (32). In older people, gradual withdrawal of psychotropic medication reduced the rate of falls (13), and this approach may also be effective in patients with RA. High fatigue levels are common in adults with RA and have been linked to pain and depression (21–33); however, there is some evidence that fatigue levels fall with disease-modifying antirheumatic drugs (DMARDs) and anti–tumor necrosis factor therapy (34–35). Swollen and tender lower extremity joints may be improved through good multidisciplinary management of the patient. Drug management of RA to reduce swollen and tender joints is complex and includes the use of DMARDs, steroids, and biologic agents. From this study, the use of steroids was associated with an increased risk of falls, and for these reasons and due to their long-term effects, it is recommended that they are used with caution.

Poor balance and lower extremity strength were significantly associated with an increased risk of falling as observed by previous RA studies (2,3). Specific exercises adapted from a research-based falls prevention program could be used to improve muscle strength and balance in adults with RA and may reduce the risk of falls (36). Exercise has been shown to reduce fatigue in adults with RA, and may also improve depression and sleeping problems (37).

Increasing HAQ disability score and high DAS28 scores were significantly associated with an increased risk of falling, as found in other studies (4–12). Fear of falling was also associated with an increased risk of falls, as found in other studies (8–9), and may result in avoidance of activities and reduction of physical ability, which could therefore increase the risk of future falls. Exercise may improve fear of falling, the functional status of the HAQ scores, and disease activity scores such as the DAS28; however, further research is needed to investigate these hypotheses (38).

In this study, the odds of falling were not significantly related to older age or female sex, which suggests that the symptoms and risk factors associated with RA override the risk factors usually associated with age and sex. This was surprising, since in the general population, adults ages >65 years, in particular women, have significantly more falls than younger adults, and there is an increased trend of falls in older ages (31,39). Hayashibara and colleagues also report that age was not associated with falls in their small prospective study of 80 women with RA (2). Older people in general are more prone to muscle weakness due to inactivity and poor gait (31). Adults of all ages with RA appear to have muscle weakness, and this may result in the similar fall rates.

Strengths of the study include its prospective, longitudinal design, high response rate, low attrition rate, and the use of validated measurement tools to collect data on fall risk factors. Attempts were made to recruit a representative sample of patients by attending a variety of outpatient clinics that included nurse-led blood monitoring sessions, primary care out of hours clinics, and rheumatology clinics. However, it is likely that patients in this study had more moderate to severe RA or more progressive disease than those generally found in primary care, and some caution should be given in applying these results to other settings. It also would have been useful to have included the foot joints in the RA disease activity assessment to investigate the contribution of swollen/tender joints within the lower extremity assessment. This may be of particular importance given that foot pain is a risk factor for falls in older people (41–42); however, a single question on whether the participant currently experienced foot pain was included in the baseline assessment due to the length of time required to assess the foot joints within limited resources. In addition, the HAQ, Short FES-I, Falls Risk Assessment Tool, VAS pain, VAS fatigue, and data on eyesight were self-reported by the participants and may be subject to errors of recall.

Adults with RA are at high risk of falls. Health professionals can identify patients of particular risk of falls by asking whether they have fallen in the past year. Patients with RA would benefit from a falls risk screening tool that utilizes the most clinically relevant and significant risk factors associated with falling. We recommend for a screening tool a 12-month history of falls, an assessment of lower extremity swollen and tender joints, an assessment of psychotropic medications, VAS fatigue and VAS pain scores, the Four-Test Balance Scale to measure postural stability, the Chair Stand Test to measure lower extremity strength, the Short FES-I to measure fear of falling, and the HAQ to measure functional ability. Future research should consider a falls prevention program that incorporates exercises that specifically target lower extremity muscle strength and challenge balance alongside a review of medication, in particular the use of psychotropic medications.

## AUTHOR CONTRIBUTIONS

All authors were involved in drafting the article or revising it critically for important intellectual content, and all authors approved the final version to be published. Dr. Stanmore had full access to all of the data in the study and takes responsibility for the integrity of the data and the accuracy of the data analysis.

**Study conception and design.** Stanmore, Oldham, Skelton, O'Neill, Campbell, Todd.

**Acquisition of data.** Stanmore, Todd.

**Analysis and interpretation of data.** Stanmore, Oldham, Skelton, O'Neill, Pilling, Campbell, Todd.
